# Copper-64-Labeled 1C1m-Fc, a New Tool for TEM-1 PET Imaging and Prediction of Lutetium-177-Labeled 1C1m-Fc Therapy Efficacy and Safety

**DOI:** 10.3390/cancers13235936

**Published:** 2021-11-25

**Authors:** Judith Anna Delage, Silvano Gnesin, John O. Prior, Jacques Barbet, Patricia Le Saëc, Séverine Marionneau-Lambot, Sébastien Gouard, Michel Chérel, Mickael Bourgeois, Niklaus Schaefer, David Viertl, Julie Katrin Fierle, Steven Mark Dunn, Alain Faivre-Chauvet

**Affiliations:** 1 Radiopharmacy Unit, Department of Pharmacy, Lausanne University Hospital and University of Lausanne, CH-1011 Lausanne, Switzerland; 2 Institute of Radiation Physics, Lausanne University Hospital and University of Lausanne, CH-1011 Lausanne, Switzerland; silvano.gnesin@chuv.ch; 3 Department of Nuclear Medicine and Molecular Imaging, Lausanne University Hospital and University of Lausanne, CH-1011 Lausanne, Switzerland; niklaus.schaefer@chuv.ch; 4 GIP Arronax, F-44800 Saint-Herblain, France; jacques.barbet@univ-nantes.fr (J.B.); mickael.bourgeois@univ-nantes.fr (M.B.); 5 CRCINA, Inserm, CNRS, CHU Nantes, University of Nantes, F-44000 Nantes, France; patricia.lesaec@univ-nantes.fr (P.L.S.); severine.marionneau-lambot@univ-nantes.fr (S.M.-L.); sebastien.gouard@univ-nantes.fr (S.G.); michel.cherel@univ-nantes.fr (M.C.); alain.faivre-chauvet@univ-nantes.fr (A.F.-C.); 6 Translational Radiopharmaceutical Sciences, Department of Nuclear Medicine and Molecular Imaging, Lausanne University Hospital and University of Lausanne, CH-1011 Lausanne, Switzerland; david.viertl@chuv.ch; 7 In Vivo Imaging Facility, Department of Research and Training, University of Lausanne, CH-1005 Lausanne, Switzerland; 8 LAbCore, Ludwig Institute for Cancer Research, Lausanne University Hospital and University of Lausanne, CH-1066 Epalinges, Switzerland; julie.fierle@unil.ch (J.K.F.); steven.dunn@chuv.ch (S.M.D.)

**Keywords:** theranostic, tumor endothelial marker 1, DOTA conjugation, copper-64, PET imaging, Lutetium-177, dosimetry

## Abstract

**Simple Summary:**

The prevalence of TEM-1 in the vasculature and the stroma of solid tumors and in malignant cells of sarcomas suggests that targeting TEM-1 could have therapeutic benefit. In this context, an anti-TEM-1 companion diagnostic may assist in the personalized medicine approach, whereby TEM-1 expression is exploited as a biomarker to select patients that would most benefit from a treatment directed toward the TEM-1 antigen. In our previous works, we have selected 1C1m-Fc, a fusion protein antibody, radiolabeled it with ^177^Lu and demonstrated that [^177^Lu]Lu-1C1m-Fc has interesting therapeutic performance. To define a suitable radiopharmaceutical companion for theranostic applications, ^64^Cu was chosen to radiolabel the fusion protein antibody. The aim of this work was thus to determine if [^64^Cu]Cu-1C1m-Fc can be considered for TEM-1 PET imaging and to predict the dosimetry of the [^177^Lu]Lu-1C1m-Fc companion therapy.

**Abstract:**

1C1m-Fc, a promising anti-TEM-1 DOTA conjugate, was labeled with ^64^Cu to target cancer cells for PET imaging and predicting the efficacy and safety of a previously studied [^177^Lu]Lu-1C1m-Fc companion therapy. DOTA-conjugated 1C1m-Fc was characterized by mass spectrometry, thin layer chromatography and immunoreactivity assessment. PET/CT and biodistribution studies were performed in human neuroblastoma xenografted mice. Absorbed doses were assessed from biodistribution results and extrapolated to ^177^Lu based on the [^64^Cu]Cu-1C1m-Fc data. The immunoreactivity was ≥ 70% after 48 h of incubation in serum, and the specificity of [^64^Cu]Cu-1C1m-Fc for the target was validated. High-resolution PET/CT images were obtained, with the best tumor-to-organ ratios reached at 24 or 48 h and correlated with results of the biodistribution study. Healthy organs receiving the highest doses were the liver, the kidneys and the uterus. [^64^Cu]Cu-1C1m-Fc could be of interest to give an indication of ^177^Lu dosimetry for parenchymal organs. In the uterus and the tumor, characterized by specific TEM-1 expression, the ^177^Lu-extrapolated absorbed doses are overestimated because of the lack of later measurement time points. Nevertheless, 1C1m-Fc radiolabeled with ^64^Cu for imaging would appear as an interesting radionuclide companion for therapeutic application with [^177^Lu]Lu-1C1m-Fc.

## 1. Introduction

Theranostics is an emerging strategy combining diagnosis and therapy to achieve personalized treatments. Currently, this approach involves a number of scientific disciplines but is particularly linked to nuclear medicine [1]. Indeed, radionuclide imaging offers the opportunity to select patients, monitor therapy, and optimize the dosimetry to increase the efficacy and safety of targeted radionuclide therapy [2]. This approach is made possible by the discovery of biomarkers overexpressed in oncologic diseases that can be used as molecular targets [3] and by the development of vectors specifically binding these targets.

In clinical practice, the same vector may be radiolabeled with two different radionuclides, one for single photon emission computed tomography (SPECT) or positron emission tomography (PET) (respectively gamma or positron emitters) and one for therapy (electron or alpha particle emitter). Currently, PET is considered as having a higher sensitivity and a better spatial resolution than SPECT and a true potential for accurate quantitative imaging [4]. While electron emitters have been used in the vast majority of clinical applications, the use of radionuclides emitting alpha particle is also emerging. Small molecules, peptides or antibodies can be chosen as vectors [5].

Tumor endothelial marker 1 (TEM-1), also referred as CD248 or endosialin, is a cell surface transmembrane protein belonging to the C-lectin receptor superfamily. TEM-1 is expressed on pericytes and fibroblasts during tissue development, tumor neovascularization and inflammation [6,7,8]. TEM-1 has been described as an interesting target, as it is expressed by tumor stroma and tumor vessels in several oncological disease but has no or limited expression in normal adult tissues [9,10]. Furthermore, a high level of TEM-1 expression is associated with a poor prognosis and correlates with the aggressiveness of the tumor [11,12]. TEM-1 was thus considered by the research community as a potential therapeutic target, and several anti-TEM-1 antibodies have been developed [13]. Antibody drug conjugates [14,15], ScFv-Fc fragments for optical imaging and radionuclide imaging [16,17,18] and a radiolabeled humanized anti-TEM-1 monoclonal antibody MORAb-004 [19,20] have been tested preclinically. Currently, no clinical trial has been conducted with radiopharmaceuticals targeting TEM-1. Nevertheless, the naked MORAb-004 antibody has been evaluated in clinical immunotherapy trials [21,22,23]. Based upon a Phase I study conducted in sarcoma patients, this compound received FDA orphan drug designation for sarcoma [6]. 

Even though radioimmunoconjugates have been studied for more than 30 years, both for hematological disorders and solid tumors [24], only one of them, [^90^Y]Ibritumomab-tiuxetan, Zevalin^®^, has been approved for the treatment of recurrent or refractory non-Hodgkin’s lymphomas [25]. Several strategies are currently used to improve radioimmunotherapy success such as antibody engineering, development of new bifunctional chelating agent (BFCA) or a choice of the most appropriate radionuclide. Radionuclide physical properties, and particularly their radioactive decay half-life, must be compatible with the radioimmunoconjugate targeting, disposition kinetics [26] and intended use. 

In our previous works, 1C1m-Fc, a novel anti-TEM-1 ScFv-Fc construct, which binds both murine and human TEM-1, has been conjugated to a BFCA, p-SCN-Bn-DOTA, radiolabeled with ^177^Lu and evaluated in a TEM-1-positive tumor model in mice. The results of the first experiments were promising, as a specific uptake of [^177^Lu]Lu-1C1m-Fc in TEM-1-positive tumor was observed [27]. The impact of DOTA conjugation was evaluated. Using a pharmacokinetic model, the number of DOTA per fusion protein antibody was demonstrated to have an impact on the pharmacokinetics and immunoreactivity of [^177^Lu]Lu-1C1m-Fc [28]. The biodistribution and imaging contrast was improved by decreasing the number of chelating agents per 1C1m-Fc molecule: one DOTA per 1C1m-Fc gave the best tumor-to-liver ratio, but the specific activity was only 200 MBq/mg. Three to four DOTA per 1C1m-Fc was also adequate and provided a higher specific activity of 400 MBq/mg.

The aim of the present work is to pursue the evaluation of this new fusion protein antibody, 1C1m-Fc, in a theranostic strategy using ^64^Cu for pretherapeutic PET imaging and dosimetry assessment and ^177^Lu for targeted radionuclide therapy. ^64^Cu presents a particular interest [29]. Indeed, this radionuclide is produced by cyclotrons, has a half-life of 12.7 h that allows PET immunoimaging and decays by emitting both low energy positrons and electrons. Promising results have already been obtained in preclinical and clinical trials using ^64^Cu as a theranostic imaging agent [30].

In this study, although the vector 1C1m-Fc is kept constant, ^64^Cu and ^177^Lu differ by their half-life, their stability in DOTA complexes and possibly by their biodistribution. We have thus studied preclinically the [^64^Cu]Cu-1C1m-Fc compound to determine if it can be considered as a new tool for TEM-1 PET imaging and to predict the dosimetry of the [^177^Lu]Lu-1C1m-Fc companion therapy.

## 2. Materials and Methods

### 2.1. Cell Lines

The human fibrosarcoma HT-1080 (TEM-1-negative) and human neuroblastoma SK-N-AS (TEM-1-positive) were acquired from American Type Culture Collection (ATCC, Manassas, VA, USA).

The two cell lines were cultured in DMEM media (Thermo Fisher Scientific, Waltham, MA, USA) supplemented with 10% fetal bovine serum (FBS, Thermo Fisher Scientific, Waltham, MA, USA), 1% penicillin/streptomycin (Thermo Fisher Scientific, Waltham, MA, USA), 0.1 mM of nonessential amino acids (Thermo Fisher Scientific, Waltham, MA, USA) were also added for SK-N-AS culture. Cells were incubated in a flask at 37 °C in a humidified atmosphere at 5% CO_2_.

### 2.2. Fusion Protein Antibody

A complete description of the 1C1m-Fc fusion protein antibody may be found in Fierle et al. [31] and in Delage et al. [27]. Briefly, this single-chain variable fragment (scFv) fused to a human Fc domain (Ig G) binds both human and murine TEM-1 with an affinity of 1 and 6 nM, respectively.

### 2.3. Conjugation

Antibody concentrations were measured at 280 nm using a spectrophotometer (NanoDrop Lite, Thermo Fisher Scientific, Waltham, MA, USA). 1C1m-Fc was conjugated with p-SCN-Bn-DOTA (Macrocyclics, Plano, TX, USA). A calculated volume of p-SCN-Bn-DOTA (25.9 mg/mL, 47 µmol/mL, in DMSO 10% *v/v*) was added to 1C1m-Fc (1 mg, 9.4 nmol) in 0.2 M carbonate buffer pH 9.0. The solution was maintained at 37 °C for 1 h. The number of DOTA conjugated per fusion protein antibody ratio was 3 to 4 DOTA. Conjugated antibodies were washed by four rounds of ultrafiltration in 0.1 M sodium acetate buffer pH 5.0 (Alfa Aesar, Haverhill, MA, USA). High pressure liquid chromatography (HPLC) was performed to assess the integrity of the conjugates. DOTA-conjugated 1C1m-Fc was subsequently stored between 2 and 8 °C. The purity and the stability of the conjugate were evaluated by HPLC, as described in Delage et al. [27].

### 2.4. Characterization of the Immunoconjugates: Mass Spectrometry Analysis

The number of chelate per antibody was determined by mass spectrometry (MS) analysis using a Q Exactive HF Orbitrap (Thermo Fisher Scientific, Waltham, MA, USA), as previously described in Delage et al. [27,28].

After analysis, the mass spectrometry spectra were deconvoluted, and the drug-to-antibody ratio (DAR) was obtained using the formula: Σ(*n* × Int)/Σ(Int), where *n* is the number of attached molecules for each peak, and Int the intensity of the peak.

### 2.5. Radiolabeling 

^64^Cu dichloride (^64^CuCl_2_) in 0.1 N HCl solution was produced by the ARRONAX cyclotron (Saint Herblain, France). A calculated volume of sodium acetate 2.5 M metal-free (Alfa Aesar, Haverhill, MA, USA) was first added to the ^64^CuCl_2_ solution, followed by a calculated volume of 5 mg/mL DOTA-conjugated 1C1m-Fc in acetate buffer 0.1 M. After 30 min incubation at 42 °C, 1 mM EDTA pH 7.0 (Sigma–Aldrich, St. Quentin Fallavier, France) was added to obtain a final concentration of 0.01 mM to complex free ^64^Cu(II).

The radiochemical purity of [^64^Cu]Cu-1C1m-Fc was determined by instant thin layer chromatography (iTLC). The release criterium was ≥95%. iTLC analyses were performed using dried iTLC-SG glass microfiber chromatography paper impregnated with silica gel (Agilent Technologies, Folsom, CA 95630). Citrate buffer (0.1 M, pH 4.5) was used as eluent. In this system, [^64^Cu]Cu-1C1m-Fc remains at Rf = 0, while unbound [^64^Cu]Cu-EDTA migrates to the solvent front (Rf = 1).

The radiochemical purity after antibody radiolabeling was assessed by iTLC-SG just after radiolabeling and 24 h after.

### 2.6. In Vitro Studies: Radio-Immunoreactivity

Immunoreactivity of [^64^Cu]Cu-1C1m-Fc was assessed using Pierce™ Streptavidin Magnetic Beads (Thermo Fischer Scientific, Waltham, MA, USA). Streptavidin is covalently coupled to the surface of the magnetic beads. For each streptavidin molecule on the bead, around 3 biotin-binding sites are available. The Streptavidin beads (250 µL, 10 mg/mL) were first coupled to biotin-conjugated TEM-1 antigen (50 µL, 0.33 mg/mL) obtained from the LAbCore immunoglobulin discovery and engineering facility, Ludwig Institute for Cancer Research, Lausanne, (following the instructions given by Thermo Fischer Scientific). The biotinylated fragment comprises the 353 N-terminal amino acids of the mature human TEM-1 protein. The TEM-1-coated beads (10 µL at 10 mg/mL) were mixed to 0.016 pmol of radiolabeled antibody in human serum, and PBS/BSA 0.1% was added to obtain a final volume of 100 µL. The mixture was incubated at 37°C in human serum for 48 h. The time points were chosen to correlate with those of the biodistribution study (until 24 h for the [^64^Cu]Cu-3DOTA-1C1m-Fc and 48 h for the [^64^Cu]Cu-4DOTA-1C1m-Fc).

The radioactivity bound to the beads, collected with a magnetic stand and, remaining in the supernatant, was measured with a gamma counter (AMG Automatic Gamma Counter, Hidex, Turku, Finland). Each test was run in duplicate. The immunoreactivity was calculated as the ratio between the activity of the beads to the total activity. Nonspecific uptake on the tube was also measured and accounted for.

### 2.7. In Vivo Characterization Studies

#### 2.7.1. Animal Model

The in vivo studies were carried out in female BALB/c nude mice from 7 to 9 weeks old (Janvier Labs, Le Genest-Saint-Isle, France or Charles River Laboratories, Wilmington, MA, USA). A group of mice was xenografted subcutaneously in the left flank with 3 × 10^6^ SK-N-AS (TEM-1-positive) cells suspended in a solution of 100 µL of medium (group 1, *n* = 14). As a negative control, a second group (group 2, *n* = 3), was grafted in the right flank with 3 × 10^6^ SK-N-AS cells and with 3 × 10^6^ HT-1080 (TEM-1-negative) cells suspended in 100 µL of a solution containing 1:1 mixture of Matrigel and medium in the left flank.

#### 2.7.2. PET Imaging Study

All mice were anesthetized for the duration of the imaging sequence by inhalation of isoflurane 2%/O_2_ and warmed on a heating pad during the scan.

For two SK-N-AS tumor-bearing mice of group 1, 10-, 20- or 30-min images (energy window 358–664 keV) were acquired on a small-animal PET/SPECT/CT device (Albira, Bruker BioSpin MRI GmbH, Ettlingen, Germany) at three time-points (respectively, 4, 24, and 48 h) after intravenous injection of 6.0 ± 0.3 MBq [^64^Cu]Cu-1C1m-Fc (50 μg of total antibody per mouse). Images were reconstructed using a three-dimensional maximum likelihood expectation maximization algorithm with 12 iterations, without postreconstruction smoothing. The PET in-plane FOV size was 80 mm with axial extension of 149 mm; reconstructed image voxel size was 0.5 mm isotropic in space. Dead-time, scatter and random corrections were applied. Coregistered CT (0.4 mA, 35 kV, 600 projections, 125 µm voxel size) was used for anatomical localization of uptake. PET and CT images were visualized and analyzed using PMOD (PMOD Technologies, version 3.7, Zurich, Switzerland).

For group 2, ten-minute images (energy window 250–750 KeV, FOV size 90 mm) of mice (*n* = 3) bearing both SK-N-AS and HT 1080 tumors were recorded 24 h after injection of 7.7 ± 0.2 MBq of [^64^Cu]Cu-1C1m-Fc (50 μg of total antibody per mouse) with an IRIS PET/CT, (Inviscan SAS, Strasbourg, France). The CT acquisition parameters were 20 s, 0.9 mA, 80 KV, 576 projections, 160 µm voxel size. PET images were reconstructed with 3D-OSEM-MC, eight subsets, eight iterations with decay, random and dead-time corrections. For CT, filtered back-projection algorithm with beam hardening and ring artefact correction was used.

#### 2.7.3. Biodistribution Study

[^64^Cu]Cu-1C1m-Fc was injected in the lateral tail vein of the mice. Animals received [^64^Cu]Cu-4DOTA-1C1m-Fc (group 1) or [^64^Cu]Cu-3DOTA-1C1m-Fc (group 2) and unlabeled 1C1m-Fc corresponding to a total antibody dose of 50 µg per mice, in a total volume of 100 µL. The average weight of animals was 17.79 ± 0.66 mg for group 1 and 17.93 ± 2.57 mg for group 2. The dose of 50 µg (470 pmol) of antibody has been selected from our previous study [27].

In group 1, animals were euthanized and exsanguinated at 4, 24, 48 h (corresponding precisely to 4.3, 26.0 and 50.2 h), after injection of the radiolabeled product and 24 h after injection for group 2. Blood was collected, and organs and tumors were removed, weighed, and counted with a gamma counter (AMG Automatic Gamma Counter, Hidex, Turku, Finland). Results were expressed as the percentage of injected activity (IA) per gram of tissue (%IA/g).

#### 2.7.4. Murine Dosimetry

Estimated absorbed doses to organs were based on the biodistribution results on SK-N-AS-bearing mice. Considered source organs for the dosimetry study were the liver, the kidneys, the lungs, the spleen, the heart content, the stomach, the small intestine, the colon, the uterus and ovaries, the tumor and the remainder of the body. 

The biodistribution for the remainder tissues was obtained by multiplying the rest-of-body mass (17.8 g average mouse mass—sum of the masses of all other considered source organs) by the normalized mass-activity concentration (g^−1^) measured in the muscle, which was taken as representative of the background body uptake.

For each mouse at each time point (4.3, 26.0 and 50.2 h), the activity in each source organ and the remainder was normalized by the total injected activity to obtain the normalized injected activity (nA). For each source organ at each time point, an average nA value was obtained ± SD. The source organs’ normalized time-activity curves (nTACs) were fitted with monoexponential functions using the kinetic module of OLINDA/EXM 2.1 (HERMES Medical Solution AB, Stockholm, Sweden). For source organs having an effective decay constant (λ_eff_) larger that the physical decay constant of ^64^Cu (λ_p,Cu64_), time-integrated activity coefficients (TIACs) were derived by monoexponential analytical time integration (extended to infinite) of fitted source organ nTACs obtained with the average nA, nA + SD and the nA–SD values, respectively. To avoid unrealistic TIACs overestimates, when the source organ λ_eff_ was smaller than the λ_p,Cu-64_, monoexponential time integration with λ_eff_ was applied only from time zero until the time of the last biokinetic measurement (50.2 h), and a monoexponential analytic integration using λ_p,Cu64_ was applied beyond (t > 50.2 h). Finally, the source organ TIACs were entered into the OLINDA/EXM 2.1 software kinetic module for organ absorbed dose estimates, where the 25 g murine model was adjusted to match the source organ average masses obtained from the mice population used in our experiment. In this process, the TIAC of the uterus and the ovaries were part of the remainder of the body. A specific absorbed dose estimate was performed for the uterus, the ovaries and the tumor. These tissues exhibited an important specific tracer uptake but were not among the source/target organs available in the murine model of the OLINDA/EXM 2.1 software. Absorbed dose estimates were thus obtained using the sphere model of OLINDA/EXM 2.1, where the average organ TIACs and the average organ masses for these reproductive organs and the tumor were applied.

#### 2.7.5. Dose Extrapolation to the ^177^Lu Compound 

To allow the comparison with previously published data for similar molecules labeled with ^177^Lu, we extrapolated the organ absorbed doses for the ^177^Lu radiolabeled compound from experimental data obtained for the [^64^Cu]Cu-1C1m-Fc murine biodistribution data. nA values for ^177^Lu were extrapolated from ^64^Cu measured data points by the application of a scale factor (SF):

$$SF\left(t_{m}\right)=exp\left(-\lambda_{p,Lu-177}\times t_{m}\right)/exp\left(-\lambda_{p,Cu-64}\times t_{m}\right)$$

where t_m_ indicates the measured time points (4.3, 26 and 50.2 h postinjection, respectively); therefore, nALu-177(t_m_) = nACu-64(t_m_) × SF(t_m_). The rescaling procedure compensates for the different physical half-life of the two radioisotopes, assuming the same biological half-life. To perform ^177^Lu nTACs’ time integration and absorbed dose estimates, we applied the methodology previously described above for the ^64^Cu.

### 2.8. Statistical Analysis

The data are expressed as mean ± SD (standard deviation). Significant differences between means were analyzed by an unpaired, 2-tailed Student *t*-test with a correction for multiple comparison using the Holm–Sidak method (α = 0.05). Statistical analyses were conducted using Prism 8.0 (GraphPad Software, San Diego, CA, USA). 

## 3. Results

### 3.1. Conjugation

1C1m-Fc conjugated with DOTA was analyzed by mass spectrometry, and the mass obtained for the native antibody was 108 394. The samples used for radiolabeling were conjugated with 3 (Figure S1a) to 4 DOTA (Figure S1b).

The conjugates were evaluated by HPLC, and the purity was, respectively, of 96.2% for 3DOTA-1C1m-Fc and 95.0% for 4DOTA-1C1m-Fc.

### 3.2. Radiolabeling

**Figure 1:**
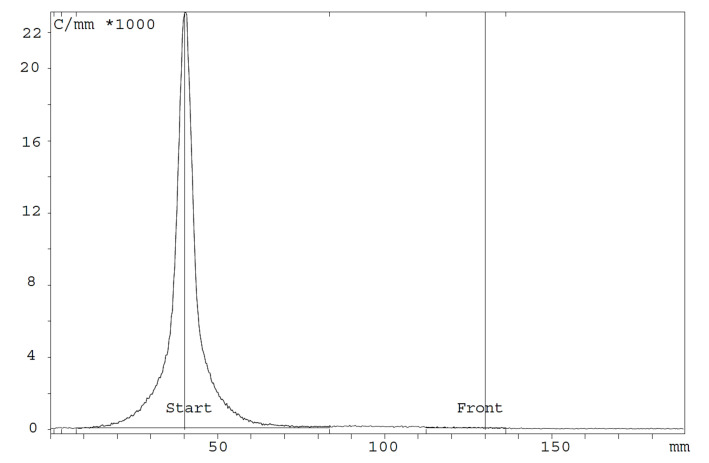
Radio–TLC example of [^64^Cu]Cu-1C1m-Fc. The radiochemical purity (RCP) is 99.85%. [^64^Cu]Cu-1C1m-Fc remains at Rf = 0, and the unbound [^64^Cu]Cu-EDTA migrates to the solvent front. With the used radiolabeling process, the average radiochemical purity was 99.5 ± 0.6% immediately after radiolabeling (*n* = 5) and 98.3 ± 2.1% (*n* = 3) after 24 h. The specific activity was comprised between 156 and 200 MBq/mg.

The release criterium for the radiochemical purity (RCP) evaluated by iTLC was more than 95% (Figure 1). 

### 3.3. In Vitro Studies: Radio-Immunoreactivity

The results of the immunoreactivity assessment of [^64^Cu]Cu-1C1m-Fc in serum media are reported in Table 1.

### 3.4. Imaging Study

**Figure 2:**
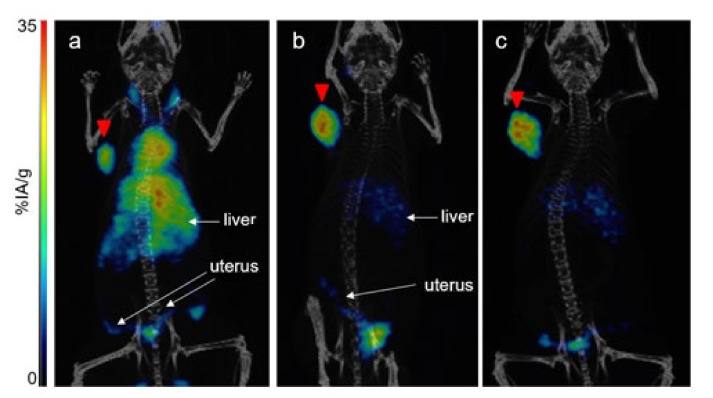
[^64^Cu]Cu-1C1m-Fc dorsal view PET/CT fusion maximum intensity projection in mouse bearing TEM-1-positive tumors (SK-N-AS, left flank, red arrow), (**a**) at 4 h, (**b**) at 24 h, (**c**) at 48 h.

High-quality PET/CT images were obtained at 4 h, 24 h and 48 h in mice bearing TEM-1-positive tumor. At 4 h, the activity was found predominantly in blood; the heart and carotid arteries were visible, as well as the liver, but the tumor was also already clearly visible. The circulating and liver activities decreased thereafter, and tumor-to-liver ratios determined by imaging were, respectively, 0.8, 1.8 and 1.7 at 4 h, 24 h and 48 h (Figure 2).

**Figure 3:**
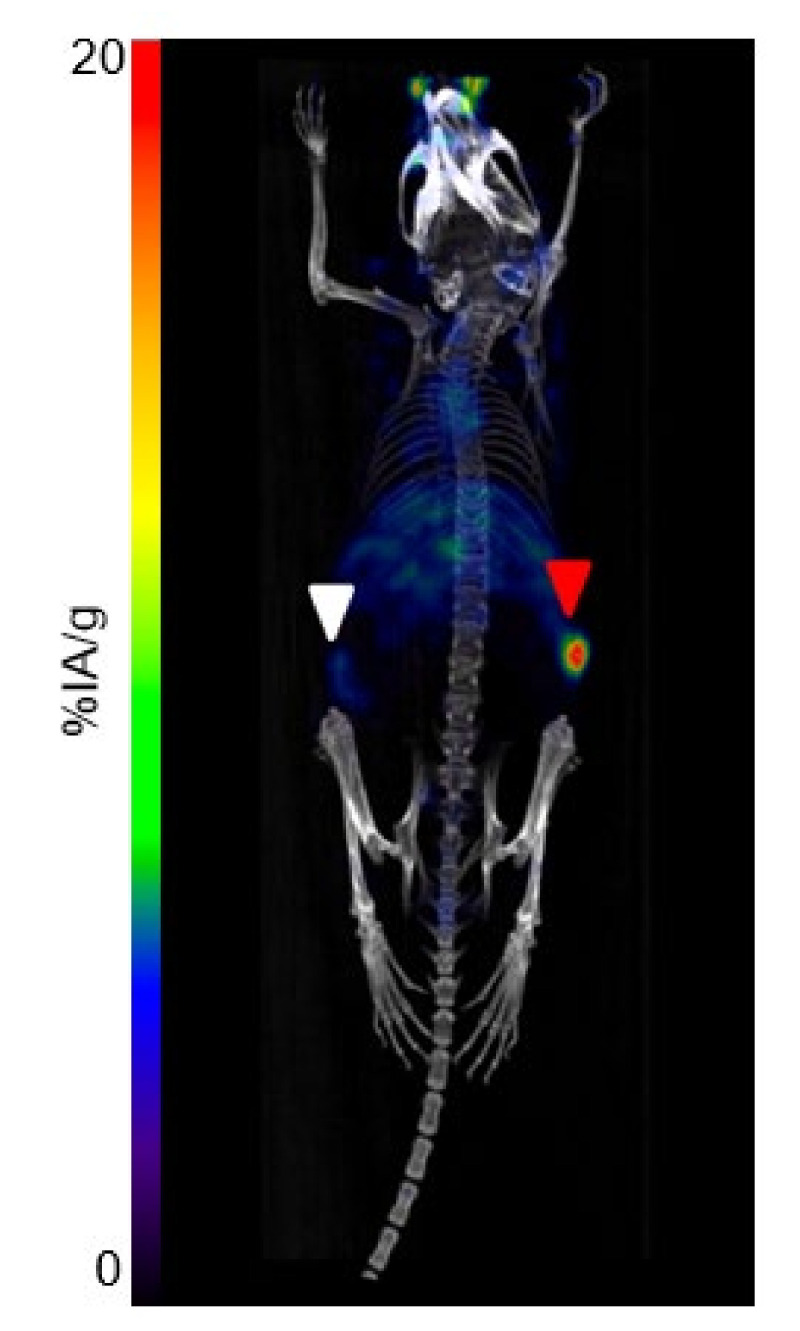
[^64^Cu]Cu-1C1m-Fc dorsal view PET/CT fusion maximum intensity projection at 24 h on mouse bearing TEM-1-negative tumor (HT-1080; left flank; white arrow) and TEM-1-positive tumor (SK-N-AS; right flank; red arrow).

The PET/CT performed at 24 h in mice (*n* = 3) bearing both TEM-1-positive and negative tumors confirmed the specificity of the uptake in TEM-1-positive tumor (higher uptake) as opposed to control tumor (lower uptake) (Figure 3).

### 3.5. Biodistribution Study

A biodistribution study of [^64^Cu]Cu-1C1m-Fc was performed in mice bearing TEM-1-positive tumors (group 1) and in mice bearing both TEM-1-positive and negative tumors as control (group 2). 

**Figure 4:**
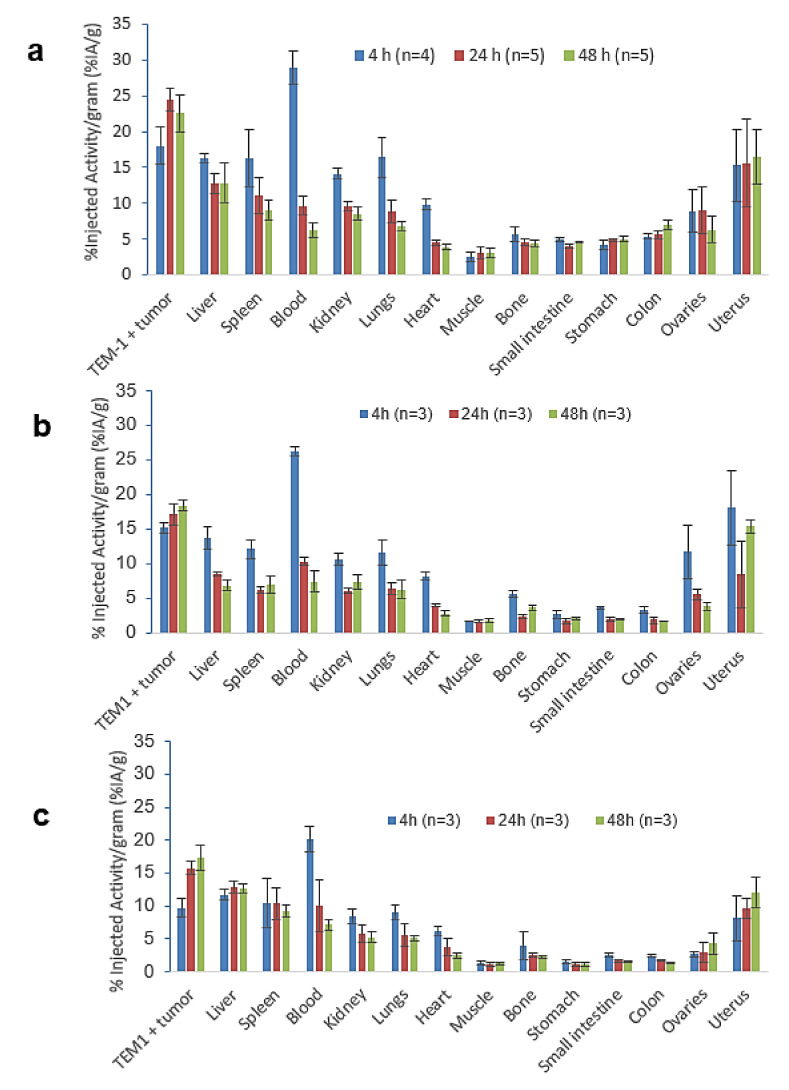
Biodistribution in BALB/c nude mice bearing TEM-1-positive tumor of (**a**) [^64^Cu]Cu-4DOTA-1C1m-Fc, group 1; (**b**) [^177^Lu]Lu-1DOTA-1C1m-Fc (data from Delage et al. [28]; (**c**) [^177^Lu]Lu-3DOTA-1C1m-Fc (data from Delage et al. [27]). The total antibody dose used for this biodistribution was 50 µg. Data are shown as mean ± SD.

For group 1 (Figure 4a), the uptake in the tumor was maximum at 24 h (24.5 ± 1.5% IA/g). The uptake in the liver decreased between 4 h (16.3 ± 0.6% IA/g) and 24 h (12.7 ± 1.4% IA/g) and remained stable until 48 h (12.8 ± 2.8% IA/g). A rapid blood clearance was observed as the amount of radiotracer in the blood decreased from 29.0 ± 2.4% IA/g at 4 h to 6.2 ± 1.1% IA/g at 48 h. The uptake in the uterus was 15.3 ± 2.9% IA/g at 4 h, and no significant difference was observed at 24 and 48 h (*p* = 0.87, unpaired Student’s *t*-test). The best tumor-to-liver ratio was observed at 24 h (1.9), and the best tumor-to-blood ratio, at 48 h (3.6) (Table 2). 

No significant difference was observed between groups 1 and 2 regarding the uptake in the TEM-1-positive tumor, the liver, the spleen or the blood (*p* = 0.93, unpaired *t*-test). For group 2, at 24 h, the uptake in TEM-1-positive tumor (21.5 ± 7.5% IA/g) was 2.4-fold higher than the one in TEM-1-negative tumor (9.0 ± 4.1% IA/g), showing the specificity of the antibody for TEM-1 (Figure S2).

The biodistribution profile of [^64^Cu]Cu-4DOTA-1C1m-Fc (Figure 4a) was very similar to the one of [^177^Lu]Lu-1DOTA-1C1m-Fc (Figure 4b, data already published in [28]) or [^177^Lu]Lu-3DOTA-1C1m-Fc (Figure 4c, data already published in [27]). The blood uptake at 4 h of [^64^Cu]Cu-4DOTA-1C1m-Fc was closer to that of [^177^Lu]Lu-1DOTA-1C1m-Fc. A hepatic accumulation was observed with the [^64^Cu]Cu-4DOTA-1C1m-Fc or [^177^Lu]Lu-3DOTA-1C1m-Fc antibody. The main difference seen was the uptake in the gastrointestinal tract; the stomach, small intestine and colon uptakes were higher with [^64^Cu]Cu-1C1m-Fc than with [^177^Lu]Lu-1C1m-Fc (*p* = 0.000012, 0.00026 and 0.00026, respectively, at 24 h, and *p* = 0.000114, 0.000002 and 0.000041 at 48 h) (unpaired Student’s *t*-test).

### 3.6. Murine Dosimetry

The organ-absorbed doses for target tissues obtained using the mouse model of the OLINDA/EXM 2.1 software are summarized in Table 3. The healthy organs receiving the most important irradiation were the liver, the uterus, the heart, the kidneys and the lungs. The estimated tumor-absorbed dose exceeded by a factor of 1.2 that of the liver.

[^177^Lu]Lu-1C1m-Fc-absorbed dose data were extrapolated from [^64^Cu]Cu-1C1m-Fc biodistribution data and compared to the experimental absorbed doses previously published in [27,28] (Table 4). Extrapolated absorbed doses for parenchymal organs (such as: liver, lung, spleen and kidneys) showed a 20% difference when compared with [^177^Lu]Lu-1DOTA-1C1m-Fc. The difference was higher with [^177^Lu]Lu-3DOTA-1C1m-Fc. In both cases, the major discrepancies were found in the gastrointestinal tract, the uterus and the tumor (extrapolated absorbed doses were overestimated).

## 4. Discussion

In the last ten years, theranostic approaches have emerged as valuable tools in oncology to identify therapeutic targets, to select the patients that would most benefit from therapeutics and to monitor the response to treatments [2,32]. Among the potential biomarkers, TEM-1 appears as an emerging target, as it is expressed in tumor vessels and in the stroma of various cancers but has no or little expression in normal adult tissues (expression is limited to endometrial stroma and occasionally fibroblast) [33,34]. Currently, the expression of TEM-1 in patients is assessed by invasive techniques such as immunohistochemistry of biopsies. In addition, these techniques do not identify the total TEM-1-positive tumor burden in a patient [20]. Consequently, immunoPET may play an important role in determining the clinical expression of tumor biomarkers.

In our previous works, we have validated the relevance of a bivalent Fc-fusion protein based on a novel single chain antibody, 1C1m-Fc, radiolabeled with ^177^Lu for a therapeutic approach [27,28]. In the present study, we studied the detection of TEM-1 in tumors using 1C1m-Fc radiolabeled with ^64^Cu for PET imaging. Another objective was to determine if [^64^Cu]Cu-1C1m-Fc can predict the dosimetry of the [^177^Lu]Lu-1C1m-Fc companion therapy.

1C1m-Fc was first conjugated to p-SCN-Bn-DOTA, as in the previous studies, with [^177^Lu]Lu-1C1m-Fc [27,28]. In a theranostic approach, the same agent, antibody and chelator must be used for imaging and therapy. The intermediate half-life of ^64^Cu (12.7 h) makes it compatible with the use of slow kinetic vectors such as antibodies. This radionuclide that emits both positrons and electrons is suitable for theranostic applications, and promising results have been obtained in recent preclinical and clinical trials [30,35,36,37,38]. DOTA-conjugated 1C1m-Fc was thus labeled with ^64^Cu, and good radiochemical purity was achieved (RCP > 95%). Cu^2+^ ions are efficiently coordinated to the DOTA ligand through four nitrogen atoms and two oxygen atoms of the pendant carboxylic groups [39,40]. The number of DOTA conjugated, determined by mass spectrometry analysis, has been shown to have an impact of the antibody biodistribution. Thus, a DOTA-to-antibody ratio between 3 and 4 was used. This ratio was selected as the lowest that allows radiolabeling of the fusion protein antibody without final purification, taking into account the variability of the copper source specific activity, while not significantly altering the biodistribution, immunoreactivity and pharmacokinetic behavior of the radioimmunoconjugates [28]. In addition, the immunoreactivity of [^64^Cu]Cu-1C1m-Fc was assessed using streptavidin-coated magnetic beads after incubation of the radiolabeled compound in serum to mimic the in vivo conditions. The immunoreactivity of [^64^Cu]Cu-1C1m-Fc evaluated in serum remained stable and ≥70% from 4 to 48 h after radiolabeling, meaning that the conjugation and the radiolabeling process do not affect the binding properties.

High-quality PET/CT images were obtained after injection of [^64^Cu]Cu-1C1m-Fc. The low energy positrons of ^64^Cu result in a good spatial resolution and a high detection rate of positive lesions [39]. Furthermore, at later time points (24 and 48 h), the relatively long half-life of ^64^Cu enables tumor assessments when higher tumor-to-background ratios are reached. The presented results motivate the use of PET/CT imaging with [^64^Cu]Cu-1C1m-Fc for the specific detection of TEM-1-positive tumors. A low but non-negligible uptake is also observed in the TEM-1-negative tumor. This may be explained by the known expression of TEM-1 in tumor neovessels and tumor stroma and by the cross-reactivity of the antibody with murine TEM-1 [41]. This visualization is allowed by the use of a fusion protein antibody that cross-reacts with both human and murine TEM-1. In this case, contrary to TEM-1-positive tumor, the antibody may not be retained by the tumor cells.

Biodistribution confirmed the imaging studies. Tumor uptake was maximum at 24 h (24.5 ± 1.5% IA/g). A specific uptake was also found in the uterus, as this endometrial stroma expresses low levels TEM-1 antigen [42]. A rapid blood clearance was observed, and the tumor-to-organ ratios were high at 24 h. The uptake in the liver decreased between 4 h and 24 h. These biodistribution data, obtained with [^64^Cu]Cu-1C1m-Fc, were compared to the previous results obtained with [^177^Lu]Lu-1C1m-Fc (conjugated with 1 or 3 DOTA), and close profiles were obtained. Nevertheless, as ^177^Lu and ^64^Cu differ in charge, in coordination geometry and number to DOTA, the biodistribution of 1Cm-Fc conjugate radiolabeled with each of these radionuclide may be slightly different [43,44]. The difference of charge between the 3 DOTA conjugates radiolabeled with ^177^Lu and ^64^Cu results in a higher uptake for the copper compound in the tumor as described in Grunberg et al. [45].

The blood uptake obtained with [^64^Cu]Cu-1C1m-Fc was closer to [^177^Lu]Lu-1DOTA-1C1m-Fc. On the contrary, the hepatic distribution (with an accumulation) of the copper-radiolabeled conjugate is closer to the one of [^177^Lu]Lu-3DOTA-1C1m-Fc. In this case, we supposed that 3 DOTA conjugate antibody (either radiolabeled with ^177^Lu or ^64^Cu) have a similar internalization into the hepatocytes. 

Compared to both ^177^Lu compounds, the % IA/g with [^64^Cu]Cu-1C1m-Fc was higher in the gastrointestinal tract (stomach, small intestine and colon). The increased uptake can be explained by the hepatobiliary excretion of ^64^Cu [46]. The absence of excretion from the liver and the gastrointestinal tract between 24 and 48 h could be explained by the transchelation of [^64^Cu^2+^] ions from the chelating agent to transport proteins [39]. DOTA–copper complexes have a good thermodynamic stability but seem to be not sufficiently inert under reducing or acidic conditions[47]. For [^64^Cu]DOTA, our group have already demonstrated that almost all the activity was associated to metalloproteins [48]. Moreover, for 1C1m-Fc, our team previously demonstrated that 44% of the total bound activity is internalized after 24 h [49]. After internalization, ^64^Cu is recycled through the transport proteins and metabolized. For ^64^Cu conversely to ^177^Lu, internalization is involved in recycling and, therefore, in nonspecific binding. Copper ions are described to have an important affinity for the endometrium [50]. Specific chelating agents such as TE1-PA [29,48] or sarcophagine (SAR) [51] have been developed that are able to form more stable copper complexes than DOTA, but, in a theranostic approach, it seems more adequate to use the very same compound for both imaging and therapy. 

The possible use of ^64^Cu-radiolabeled compounds in the imaging step to predict absorbed dose to tumors and dose-limiting organs for the same compound labeled with a therapeutic radioisotope (for instance ^177^Lu) is of great interest. Thus, absorbed doses obtained experimentally in our previous studies with [^177^Lu]Lu-1DOTA-1C1m-Fc or [^177^Lu]Lu-3DOTA-1C1m-Fc [27,28] have been compared to extrapolated ones from the present [^64^Cu]Cu-1C1m-Fc study. 

Extrapolated ^177^Lu absorbed doses (from ^64^Cu mice data) for parenchymal organs (such as: liver, lung, spleen and kidneys) provided good predictive dosimetry values (relative differences within 20%) when compared with [^177^Lu]Lu-1DOTA-1C1m-Fc. We obtained an inferior matching when comparing Lu-extrapolated organ doses with the [^177^Lu]Lu-3DOTA-1C1m-Fc (up to 80% overestimation in lung). The observed discrepancy correlates with the data obtained in the biodistribution study.

A common feature is the higher extrapolated absorbed doses in the gastrointestinal tract than can be related to the known uptake in these tissues for the ^64^Cu-radiolabeled compound compared to that of ^177^Lu, as described previously [46].

^177^Lu-absorbed dose extrapolations to the tumor and the uterus from the ^64^Cu-radiolabeled compound are higher compared to published dosimetry data for [^177^Lu]Lu-1DOTA-1C1m-Fc or [^177^Lu]Lu-3DOTA-1C1m-Fc (around +50% and +100%, respectively). This overestimation is explained by the lack of later time acquisition points after 50 h for the ^64^Cu-labeled compounds. For both the tumor and the uterus, the uptake in these TEM-1-expressing tissues is still not decreasing at 50 h postinjection (the last measured time point), and the extrapolation to infinity based on the physical decay of ^177^Lu overestimates the actual value. 

In future developments, it would be interesting to study the combination of TEM-1 and fibroblast-activated protein (FAP) targeting. Indeed, FAP inhibitors (FAPIs) target the tumor stroma, which is enriched in cancer-associated fibroblasts (CAFs), which are essential for proliferation and metastasis [52]. Contrary to FAPIs, radiolabeled 1C1m-Fc can target both microenvironment and tumor TEM-1-positive cells. Nevertheless, if the antibody quantity is too low, 1C1m-Fc could be blocked in the microenvironment, thereby reducing the quantity of anti-TEM-1 in the tumor and the antitumoral efficacy. The usage of these two approaches could thus be synergic. Similarly to 1C1m-Fc, FAPIs present an uptake in the endometrium that have to be taken into account. This uptake increases during the premenopausal and may be linked to the cyclic regeneration as FAPIs accumulate in tissue during the remodeling process [53]. 

## 5. Conclusions

In this study, we have demonstrated that [^64^Cu]Cu-1C1m-Fc permits the visualization of TEM-1 expression with high resolution PET images and allows for the selection of patients who are candidates for [^177^Lu]Lu-1C1m-Fc therapy. Furthermore, extrapolated dosimetry based on [^64^Cu]Cu-1C1m-Fc data could be used as an indicator to predict the toxicity in parenchymal organs, as increased tissues-absorbed doses were measured in TEM-1-positive tissues.

1C1m-Fc radiolabeled with ^64^Cu for imaging would appear as an interesting radionuclide companion for therapeutic application with [^177^Lu]Lu-1C1m-Fc. In perspective, this work points out the importance of future developments, such as human dosimetry extrapolation studies, aiming at the assessment of treatment safety in patients for radiolabeled compounds targeting TEM-1.

## 6. Patents

J.K.F. and S.M.D. hold patents in the domain of antibodies and, in particular, on the 1C1m antibody used in this study.

## Figures and Tables

**Table 1:** Immunoreactivity assessment of [^64^Cu]Cu-1C1m-Fc after incubation in serum. The results are expressed as mean ± SD.

Immunoreactivity (%) ± SD	4 h	24 h	48 h
[^64^Cu]Cu-3DOTA-1C1m-Fc	76 ± 1.4 (*n* = 2)	70 (*n* = 1)	NA
[^64^Cu]Cu-4DOTA-1C1m-Fc	75 ± 15 (*n* = 3)	77 ± 14 (*n* = 3)	72 ± 13 (*n* = 3)

**Table 2:** Tumor-to-organ ratio of [^64^Cu]Cu-1C1m-Fc determined by biodistribution of group 1 at 4 h, 24 h and 48 h.

Ratio	4 h	24 h	48 h
Tumor/Liver	1.1	1.9	1.8
Tumor/Spleen	1.1	2.2	2.5
Tumor/Blood	0.6	2.5	3.6
Tumor/Kidney	1.3	2.5	2.6
Tumor/Lungs	1.1	2.8	3.3
Tumor/Heart	1.8	5.4	5.8
Tumor/Muscle	7.2	8.0	7.3
Tumor/Bone	3.2	5.3	5.1
Tumor/Small Intestine	3.6	6.0	4.9
Tumor/Stomach	4.3	5.0	4.5
Tumor/Colon	3.3	4.3	3.2
Tumor/Ovaries	2.0	2.7	3.6
Tumor/Uterus	1.2	1.6	1.4

**Table 3:** Measured average organ masses and TIAC (only for source organs) and estimated organ doses obtained with the OLINDA/EXM 2.1 software for [^64^Cu]Cu-1C1m-Fc. Source organs are indicated with *. Absorbed dose estimates using the sphere model of the OLINDA/EXM 2.1 software are labeled with ^s^. The normalized time–activity curves for the considered source organs are presented in the Supplementary Materials (Figure S3).

Organ	Mean Organ Mass (g)	TIAC (MBq.h/MBq)			Absorbed Dose (mGy/MBq)		
		Mean	Mean − SD	Mean + SD	Mean	Mean − SD	Mean + SD
Brain					30	20.7	39.3
Large Intestine *	0.25	0.264	0.199	0.321	110	81.1	137
Small Intestine *	0.58	0.422	0.263	0.565	87.4	57.3	115
Stomach Wall *	0.11	0.093	0.078	0.108	98.3	78.2	118
Heart *	0.14	0.182	0.169	0.194	137	118	155
Kidneys *	0.27	0.569	0.535	0.601	167	149	184
Liver *	1.01	2.613	2.242	2.932	184	152	212
Lungs *	0.14	0.328	0.248	0.407	159	119	198
Pancreas					38.2	27.8	48.4
Skeleton					33.2	23.2	43
Spleen *	0.08	0.189	0.174	0.204	169	149	189
Testes					30.1	20.7	39.4
Thyroid					30.8	21.2	40.3
Urinary Bladder					30.6	21.1	40
Total Body	17.8	13.349	10.311	16.631	46.1	34.5	57.3
Rest of the body *	15.2	7.06	4.857	9.25			
Uterus *^,s^	0.09	0.246	0.108	0.363	178	78.4	263
Ovaries *^,s^	0.04	0.054	0.034	0.073	85.1	53.6	115
Tumor *^,s^	0.11	0.377	0.141	0.613	225	84.2	366

**Table 4:** 1: TIAC (only for source organs) and organ-absorbed doses for [^177^Lu]Lu-1C1m-Fc extrapolated from experimental murine biodistribution data of [^64^Cu]Cu-1C1m-Fc. Source organs are indicated with *. Absorbed dose estimates using the sphere model of the OLINDA/EXM 2.1 software are labeled with ^s^. 2: Absorbed doses of [^177^Lu]Lu-1DOTA-1C1m-Fc from experimental data obtained in our previous study [28]. 3: Difference ratio between the estimated absorbed dose and the absorbed dose obtained from biodistribution data of [^177^Lu]Lu-1DOTA-1C1m-Fc. 4: Absorbed doses of [^177^Lu]Lu-3DOTA-1C1m-Fc from experimental data obtained in our previous study [27]. 5: Difference ratio between the estimated absorbed dose and the absorbed dose obtained from biodistribution data of [^177^Lu]Lu-3DOTA-1C1m-Fc.

Target Organs	1: [^177^Lu]Lu-1C1m-Fc TIAC and Absorbed Dose Extrapolation from [^64^Cu]Cu-1C1m-Fc Biodistribution Data		2: [^177^Lu]Lu-1DOTA-1C1m-Fc Absorbed Dose (mGy/MBq) AD2 Data from [28]	3: Difference Ratio between AD1 and AD2 (%)	4: [^177^Lu]Lu-3DOTA-1C1m-Fc Absorbed Dose (mGy/MBq) AD3 Data from [27]	5: Difference Ratio between AD1 and AD3 (%)
	TIAC (MBq-h/MBq)	Absorbed Dose (mGy/MBq) AD1				
Brain		485	408	19		
Large Intestine *	2.921	1490	703	112	328	354
Small Intestine *	5.844	1350	577	134	438	208
Stomach Wall *	0.803	1130	1660	−32	1150	−2
Heart *	0.45	904	1110	−19	363	159
Kidneys *	1.99	1070	1320	−19	705	52
Liver *	16.559	1630	1790	−9	2230	−27
Lungs *	0.864	976	983	−1	539	81
Pancreas		512	441	16		
Skeleton		492	418	18		
Spleen *	0.672	1090	1180	−8	1200	−9
Testes		485	409	19		
Thyroid		486	409	19		
Urinary Bladder		486	534	−9		
Total Body	130.205	590	549	7		
Rest of the body *	100.102					
Uterus *^,s^	3.31	2890	1830	58	1500	93
Ovaries *^,s^	0.272	570	742	−23		
Tumor *^,s^	5.294	3850	2530	52	1820	111

## Data Availability

The data presented in this study are available in article or Supplementary Materials here.

## Supplementary Materials

The following are available online at https://www.mdpi.com/article/10.3390/cancers13235936/s1, Figure S1: Mass spectra of 1C1m-Fc conjugated with DOTA (a) DARavr = (0 × 15 + 1 × 60 + 2 × 95 + 3 × 90 + 4 × 95 + 5 × 55 + 6 × 40 + 7 × 15 + 8 × 5)/(15 + 60 + 95 + 90 + 95 + 55 + 40 + 15 + 5) = 3; (b) DARavr = (0 × 8 + 2 × 80 + 3 × 100 + 4 × 75 + 5 × 70 + 6 × 70 + 7 × 50)/(8 + 80 + 100 + 75 + 70 + 70 + 50) = 4, Figure S2: Biodistribution of [64Cu]Cu-1C1m in BALB/c nude mice bearing TEM-1-positive and negative tumor, group 2, Figure S3: Normalized time–activity curves for the considered source organs (experimental data in blue with respective ± SD interval). Red lines represent monoexponential fitting curves obtained for source organ nTACs. The coefficient of determination (R2) of the fit in respect to experimental data (blue dots) is also reported.

## References

[1] Filippi L., Chiaravalloti A., Schillaci O., Cianni R., Bagni O. (2020). Theranostic Approaches in Nuclear Medicine: Current Status and Future Prospects. Expert Rev. Med. Devices.

[2] Keinänen O., Fung K., Brennan J.M., Zia N., Harris M., van Dam E., Biggin C., Hedt A., Stoner J., Donnelly P.S. (2020). Harnessing64Cu/67Cu for a Theranostic Approach to Pretargeted Radioimmunotherapy. Proc. Natl. Acad. Sci. USA.

[3] Langbein T., Weber W.A., Eiber M. (2019). Future of Theranostics: An Outlook on Precision Oncology in Nuclear Medicine. J. Nucl. Med..

[4] Accorsi R. (2008). Brain Single-Photon Emission CT Physics Principles. Am. J. Neuroradiol..

[5] Jadvar H., Chen X., Cai W., Mahmood U. (2018). Radiotheranostics in Cancer Diagnosis and Management. Radiology.

[6] Teicher B.A. (2019). CD248: A Therapeutic Target in Cancer and Fibrotic Diseases. Oncotarget.

[7] MacFadyen J.R., Haworth O., Roberston D., Hardie D., Webster M.T., Morris H.R., Panicoc M., Sutton-Smith M., Dell A., van der Geer P. (2005). Endosialin (TEM1, CD248) is a Marker of Stromal Fibroblasts and is not Selectively Expressed on Tumour Endothelium. FEBS Lett..

[8] Tomkowicz B., Rybinski K., Foley B., Ebel W., Kline B., Routhier E., Sass P., Nicolaides N.C., Grasso L., Zhou Y. (2007). Interaction of Endosialin/TEM1 with Extracellular Matrix Proteins Mediates Cell Adhesion and Migration. Proc. Natl. Acad. Sci. USA.

[9] Simonavicius N., Robertson D., Bax D.A., Jones C., Huijbers I.J., Isacke C. (2008). Endosialin (CD248) is a Marker of Tumor-Associated Pericytes in High-Grade Glioma. Mod. Pathol..

[10] MacFadyen J., Savage K., Wienke D., Isacke C.M. (2007). Endosialin Is Expressed on Stromal Fibroblasts and CNS Pericytes in Mouse Embryos and is Downregulated during Development. Gene Expr. Patterns.

[11] Davies G., Cunnick G.H., Mansel R.E., Mason M.D., Jiang W.G. (2004). Levels of Expression of Endothelial Markers Specific to Tumour-Associated Endothelial Cells and their Correlation with Prognosis in Patients with Breast Cancer. Clin. Exp. Metastasis.

[12] O’Shannessy D.J.J., Somers E.B., Chandrasekaran L.K., Nicolaides N.C., Bordeaux J., Gustavson M.D. (2014). Influence of Tumor Microenvironment on Prognosis in Colorectal Cancer: Tissue Architecture-Dependent Signature of Endosialin (TEM-1) and Associated Proteins. Oncotarget.

[13] Maia M., Conway E. (2012). CD248: Reviewing its Role in Health and Disease. Curr. Drug Targets.

[14] Rouleau C., Gianolio D.A., Smale R., Roth S.D., Krumbholz R., Harper J., Munroe K.J., Green T.L., Horten B.C., Schmid S.M. (2015). Anti-Endosialin Antibody–Drug Conjugate: Potential in Sarcoma and Other Malignancies. Mol. Cancer Ther..

[15] Thomas A., Teicher B.A., Hassan R. (2016). Antibody–Drug Conjugates for Cancer Therapy. Lancet Oncol..

[16] Li C., Wang J., Hu J., Feng Y., Hasegawa K., Peng X., Duan X., Zhao A., Mikitsh J.L., Muzykantov V.R. (2014). Development, Optimization, and Validation of Novel anti-TEM1/CD248 Affinity Agent for Optical Imaging in Cancer. Oncotarget.

[17] Zhao A., Nunez-Cruz S., Li C., Coukos G., Siegel D.L., Scholler N. (2011). Rapid Isolation of High-Affinity Human Antibodies Against the Tumor Vascular Marker Endosialin/TEM1, Using a Paired Yeast-Display/Secretory Scfv Library Platform. J. Immunol. Methods.

[18] Cicone F., Denoel T., Gnesin S., Riggi N., Irving M., Jakka G., Schaefer N., Viertl D., Coukos G., Prior J.O. (2020). Preclinical Evaluation and Dosimetry of [(111)In]CHX-DTPA-scFv78-Fc Targeting Endosialin/Tumor Endothelial Marker 1 (TEM1). Mol. Imaging Biol..

[19] Chacko A.M., Li C., Nayak M., Mikitsh J.L., Hu J., Hou C., Grasso L., Nicolaides N.C., Muzykantov V.R., Divgi C.R. (2014). Development of 124I Immuno-PET Targeting Tumor Vascular TEM1/Endosialin. J. Nucl. Med..

[20] Lange S.E., Zheleznyak A., Studer M., O’Shannessy D.J., Lapi S.E., Van Tine B.A. (2016). Development of 89Zr-Ontuxizumab for in vivo TEM-1/endosialin PET Applications. Oncotarget.

[21] Grothey A., Strosberg J.R., Renfro L.A., Hurwitz H.I., Marshall J.L., Safran H., Guarino M.J., Kim J.P., Hecht J.R., Weil S.C. (2018). A Randomized, Double-Blind, Placebo-Controlled Phase II Study of the Efficacy and Safety of Monotherapy Ontuxizumab (MORAb-004) Plus Best Supportive Care in Patients with Chemorefractory Metastatic Colorectal Cancer. Clin. Cancer Res..

[22] Norris R.E., Fox E., Reid J.M., Ralya A., Liu X.W., Minard C., Weigel B.J. (2018). Phase 1 Trial of Ontuxizumab (MORAb-004) in Children with Relapsed or Refractory Solid Tumors: A Report from the Children’s Oncology Group Phase 1 Pilot Consortium (ADVL1213). Pediatr. Blood Cancer..

[23] Diaz L.A., Coughlin C.M., Weil S.C., Fishel J., Gounder M.M., Lawrence S., Azad N., O’Shannessy D.J., Grasso L., Wustner J. (2015). A First-in-Human Phase I Study of MORAb-004, a Monoclonal Antibody to Endosialin in Patients with Advanced Solid Tumors. Clin. Cancer Res..

[24] Bourgeois M., Bailly C., Frindel M., Guérard F., Chérel M., Faivre-Chauvet A., Kraeber-Bodéré F., Bodet-Milin C. (2017). Radioimmunoconjugates for Treating Cancer: Recent Advances and Current Opportunities. Expert Opin. Biol. Ther..

[25] Puvvada S.D., Guillen-Rodriguez J.M., Yan J., Inclan L., Heard K., Rivera X.I., Answer F., Mahadevan D., Schatz J.H., Persky D.O. (2018). Yttrium-90-Ibritumomab Tiuxetan (Zevalin (R)) Radioimmunotherapy after Cytoreduction with ESHAP Chemotherapy in Patients with Relapsed Follicular Non-Hodgkin Lymphoma: Final Results of a Phase II Study. Oncol. Basel.

[26] Caserta E., Chea J., Minnix M., Poku E.K., Viola D., Vonderfecht S., Yazaki P., Crow D., Khalife J., Sanchez J.F. (2018). Copper 64–Labeled Daratumumab as a PET/CT Imaging Tracer for Multiple Myeloma. Blood.

[27] Delage J.A., Faivre-Chauvet A., Fierle J.K., Gnesin S., Schaefer N., Coukos G., Dunn M., Viertl D.S., Prior J.O. (2020). (177) Lu Radiolabeling and Preclinical Theranostic Study of 1C1m-Fc: An Anti-TEM-1 scFv-Fc Fusion Protein in Soft Tissue Sarcoma. EJNMMI Res..

[28] Delage J.A., Faivre-Chauvet A., Barbet J., Fierle J.K., Schaefer N., Coukos G., Dunn M., Viertl D.S., Prior J.O. (2021). Impact of DOTA Conjugation on Pharmacokinetics and Immunoreactivity of [(177)Lu]Lu-1C1m-Fc, an Anti TEM-1 Fusion Protein Antibody in a TEM-1 Positive Tumor Mouse Model. Pharmaceutics.

[29] Navarro A.S., Le Bihan T., Le Saec P., Bris N.L., Bailly C., Sai-Maurel C., Bourgeois M., Chérel M., Tripier R., Faivre-Chauvet A. (2019). TE1PA as Innovating Chelator for (64)Cu Immuno-TEP Imaging: A Comparative In Vivo Study with DOTA/NOTA by Conjugation on 9E7.4 mAb in a Syngeneic Multiple Myeloma Model. Bioconjug. Chem..

[30] Mortimer J.E., Bading J.R., Colcher D.M., Conti P.S., Frankel P.H., Carroll M.I., Tong S., Poku E., Miles J.K., Shively J.E. (2014). Functional Imaging of Human Epidermal Growth Factor Receptor 2-Positive Metastatic Breast Cancer Using (64)Cu-DOTA-trastuzumab PET. J. Nucl. Med..

[31] Fierle J.K., Abram-Saliba J., Brioschi M., Detiani M., Coukos G., Dunn S.M. (2019). Integrating SpyCatcher/SpyTag Covalent Fusion Technology into Phage Display Workflows for Rapid Antibody Discovery. Sci. Rep..

[32] Liu S., Li D., Park R., Liu R., Xia Z., Guo J., Krasnoperov V., Gill P.S., Li Z., Shan H. (2013). PET Imaging of Colorectal and Breast Cancer by Targeting EphB4 Receptor with 64Cu-Labeled hAb47 and hAb131 Antibodies. J. Nucl. Med..

[33] Christian S., Winkler R., Helfrich I., Boos A.M., Besemfelder E., Schadendorf D., Augustin H.G. (2008). Endosialin (Tem1) Is a Marker of Tumor-Associated Myofibroblasts and Tumor Vessel-Associated Mural Cells. Am. J. Pathol..

[34] Christian S., Ahorn H., Koehler A., Eisenhaber F., Rodi H.-P., Garin-Chesa P., Park J.E., Rettig W.J., Lenter M.C. (2001). Molecular Cloning and Characterization of Endosialin, a C-type Lectin-like Cell Surface Receptor of Tumor Endothelium. J. Biol. Chem..

[35] Qin C., Liu H., Chen K., Hu X., Ma X., Lan X., Zhang Y., Cheng Z. (2014). Theranostics of Malignant Melanoma with ^64^CuCl_2_. J. Nucl. Med..

[36] Chakravarty R., Chakraborty S., Dash A. (2016). _64_Cu_2+_ Ions as PET Probe: An Emerging Paradigm in Molecular Imaging of Cancer. Mol. Pharm..

[37] Johnbeck C.B., Knigge U., Loft A., Berthelsen A.K., Mortensen J., Oturai P., Langer S.W., Elema D.R., Kjaer A. (2017). Head-to-Head Comparison of ^64^Cu-DOTATATE and ^68^Ga-DOTATOC PET/CT: A Prospective Study of 59 Patients with Neuroendocrine Tumors. J. Nucl. Med..

[38] Piccardo A., Paparo F., Puntoni M., Righi S., Bottoni G., Bacigalupo L., Zanardi S., DeCensi A., Ferrarazzo G., Gambaro M. (2017). 64CuCl2 PET/CT in Prostate Cancer Relapse. J. Nucl. Med..

[39] Boschi A., Martini P., Janevik-Ivanovska E., Duatti A. (2018). The Emerging Role of Copper-64 Radiopharmaceuticals as Cancer Theranostics. Drug Discov. Today.

[40] Wadas T., Wong E.H., Weisman G.R., Anderson C.J. (2010). Coordinating Radiometals of Copper, Gallium, Indium, Yttrium, and Zirconium for PET and SPECT Imaging of Disease. Chem. Rev..

[41] Dolznig H., Schweifer N., Puri C., Kraut N., Rettig W.J., Kerjaschki D., Garin-Chesa P. (2005). Characterization of Cancer Stroma Markers: In Silico Analysis of an mRNA Expression Database for Fibroblast Activation Protein and Endosialin. Cancer Immun..

[42] Opavsky R., Haviernik P., Jurkovicova D., Garin M.T., Copeland N.G., Gilbert D.J., Jenkins N.A., Bies J., Garfield S., Pastorekova S. (2001). Molecular Characterization of the Mouse Tem1/Endosialin Gene Regulated by Cell Density In Vitro and Expressed in Normal Tissues In Vivo. J. Biol. Chem..

[43] Rinne S.S., Leitao C.D., Gentry J., Mitran B., Abouzayed A., Tolmachev V., Ståhl S., Löfblom J., Orlova A. (2019). Increase in Negative Charge of 68Ga/Chelator Complex Reduces Unspecific Hepatic Uptake but does not Improve Imaging Properties of HER3-targeting Affibody Molecules. Sci. Rep..

[44] Cooper M.S., Ma M.T., Sunassee K., Shaw K.P., Williams J.D., Paul R.L., Donnelly P.S., Blower P.J. (2012). Comparison of (64)Cu-Complexing Bifunctional Chelators for Radioimmunoconjugation: Labeling Efficiency, Specific Activity, and In Vitro/In Vivo Stability. Bioconjug. Chem..

[45] Grunberg J., Jeger S., Sarko D., Dennler P., Zimmermann K., Mier W., Schibli R. (2013). DOTA-Functionalized Polylysine: A High Number of DOTA Chelates Positively Influences the Biodistribution of Enzymatic Conjugated Anti-Tumor Antibody chCE7agl. PLoS ONE.

[46] Capasso E., Durzu S., Piras S., Zandieh S., Knoll P., Haug A., Hacker M., Meleddu C., Mirzaei S. (2015). Role of 64CuCl2 PET/CT in Staging of Prostate Cancer. Ann. Nucl. Med..

[47] Cai Z., Anderson C.J. (2014). Chelators for Copper Radionuclides in Positron Emission Tomography Radiopharmaceuticals. J. Label. Compd. Radiopharm..

[48] Frindel M., Camus N., Rauscher A., Bourgeois M., Alliot C., Barré L., Gestin J.-F., Tripier R., Faivre-Chauvet A. (2014). Radiolabeling of HTE1PA: A New Monopicolinate Cyclam Derivative for Cu-64 Phenotypic Imaging. In vitro and In Vivo Stability Studies in Mice. Nucl. Med. Biol..

[49] D’Onofrio A., Gano L., Melo R., Mendes F., Oliveira M.C., Denoël T., Schaefer N., Viertl D., Fierle J., Coukos G. (2021). Biological Evaluation of New TEM1 Targeting Recombinant Antibodies for Radioimmunotherapy: In Vitro, In Vivo and in Silico Studies. Eur. J. Pharm. Biopharm..

[50] Pérez-Debén S., Gonzalez-Martin R., Palomar A., Quiñonero A., Salsano S., Dominguez F. (2020). Copper and Lead Exposures Disturb Reproductive Features of Primary Endometrial Stromal and Epithelial Cells. Reprod. Toxicol..

[51] Cai H., Li Z., Huang C.-W., Park R., Shahinian A.H., Conti P.S. (2010). An Improved Synthesis and Biological Evaluation of a New Cage-Like Bifunctional Chelator, 4-((8-amino-3,6,10,13,16,19-hexaazabicyclo[6.6.6]icosane-1-ylamino)methyl)benzoic acid, for 64Cu Radiopharmaceuticals. Nucl. Med. Biol..

[52] Shi X., Xing H., Yang X., Li F., Yao S., Zhang H., Zhao H., Hacker M., Huo L., Li X. (2021). Fibroblast Imaging of Hepatic Carcinoma with 68Ga-FAPI-04 PET/CT: A Pilot Study in Patients with Suspected Hepatic Nodules. Eur. J. Nucl. Med. Mol. Imaging.

[53] Dendl K., Koerber S.A., Finck R., Mokoala K.M.G., Staudinger F., Schillings L., Heger U., Röhrich M., Kratochwil C., Sathekge M. (2021). 68Ga-FAPI-PET/CT in Patients with Various Gynecological Malignancies. Eur. J. Nucl. Med. Mol. Imaging.

