# Supplementary material for: Copper-64-Labeled 1C1m-Fc, a New Tool for TEM-1 PET Imaging and Prediction of Lutetium-177-Labeled 1C1m-Fc Therapy Efficacy and Safety
Source: Cancers (Basel). 2021 Nov 25;13(23):5936. doi: 10.3390/cancers13235936 (PMC8657097; doi:10.3390/cancers13235936)
Supplement: Supplementary file 1 [file cancers-13-05936-s001.zip › cancers-1414785-supplementary.pdf]

cancers

Supplementary Materials

Copper-64-Labeled 1C1m-Fc, a New Tool for TEM-1 PET Imaging and Prediction of Lutetium-177-Labeled 1C1m-Fc Therapy Efficacy and Safety

Judith Anna Delage, Silvano Gnesin, John O. Prior, Jacques Barbet [4], Patricia Le Saëc, Séverine Marionneau Lambot, Sébastien Gouard, Michel Cherel, Mickael Bourgeois [4], Niklaus Schaefer, David Viertl[7], Julie Katrin Fierle, Steven Mark Dunn and Alain Faivre-Chauvet

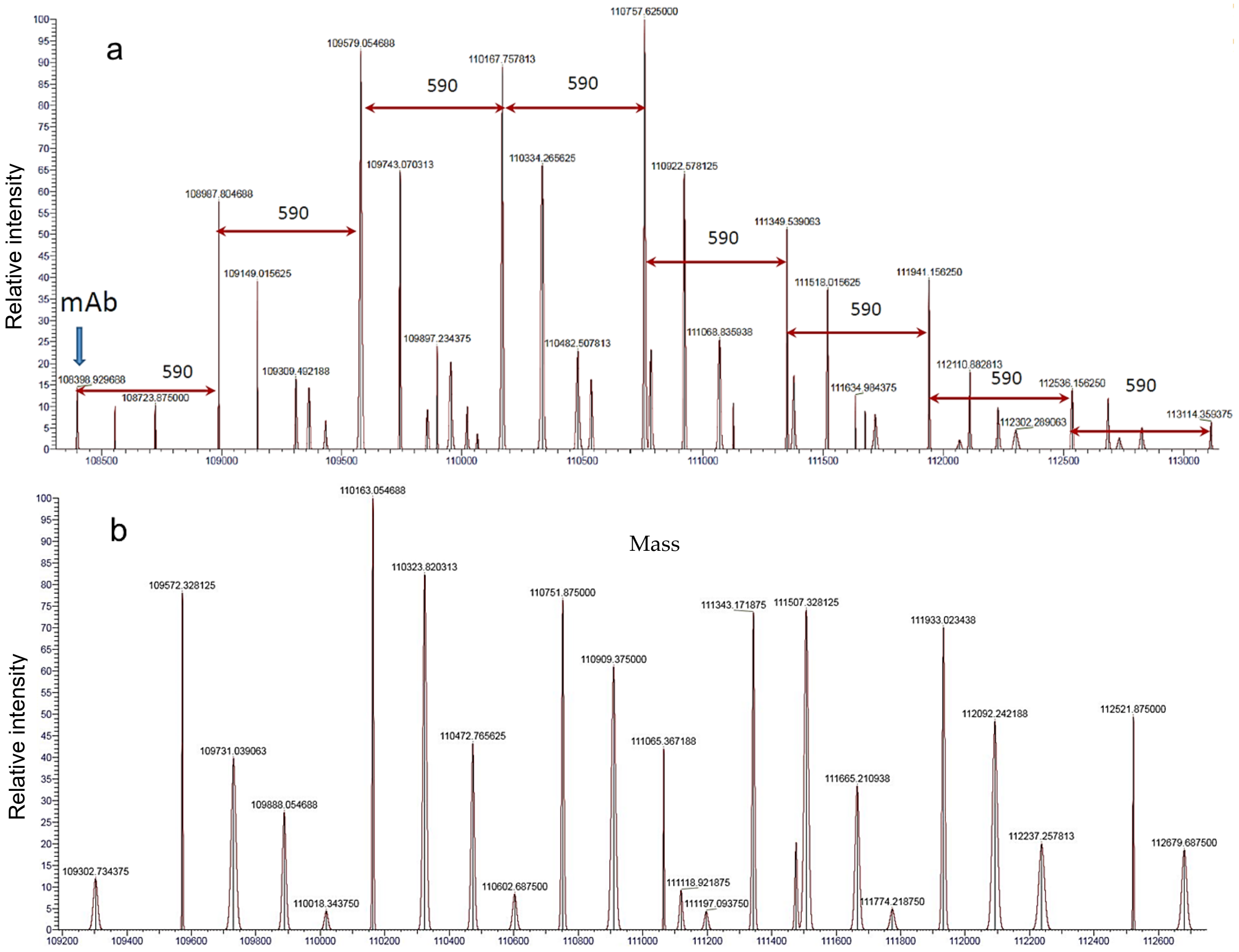

Figure S1. Mass spectra of 1C1m-Fc conjugated, with DOTA. (**a**) DARavr =(0*15+1*60+2*95+3*90+4*95+5*55+6*40+7*15+8*5) / (15+60+95+90+95+55+40+15+5) = 3; (**b**) DARavr = (0*8+2*80+3*100+4*75+5*70+6*70+7*50)/(8+80+100+75+70+70+50) = 4.

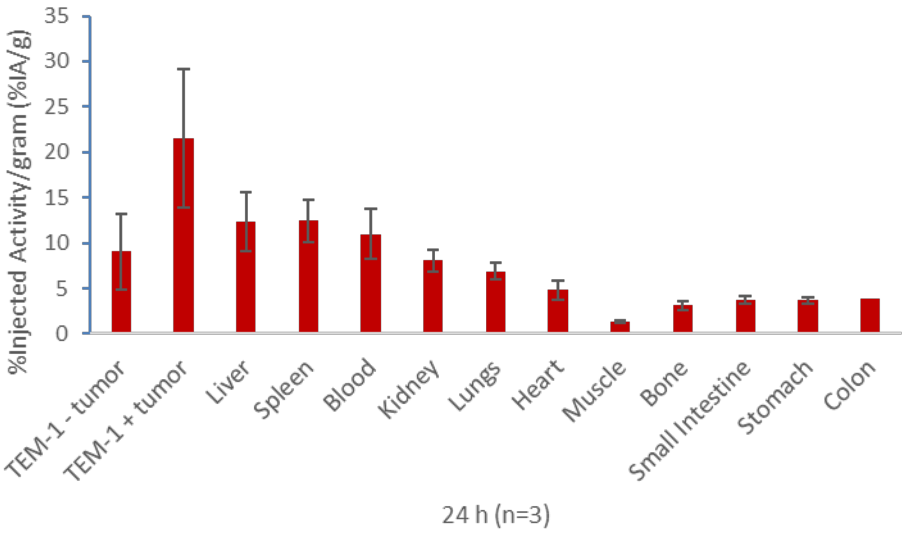

Figure S2. Biodistribution of [^{64}Cu]Cu-1C1m in BALB/c nude mice bearing TEM-1 positive and negative tumor, group 2.

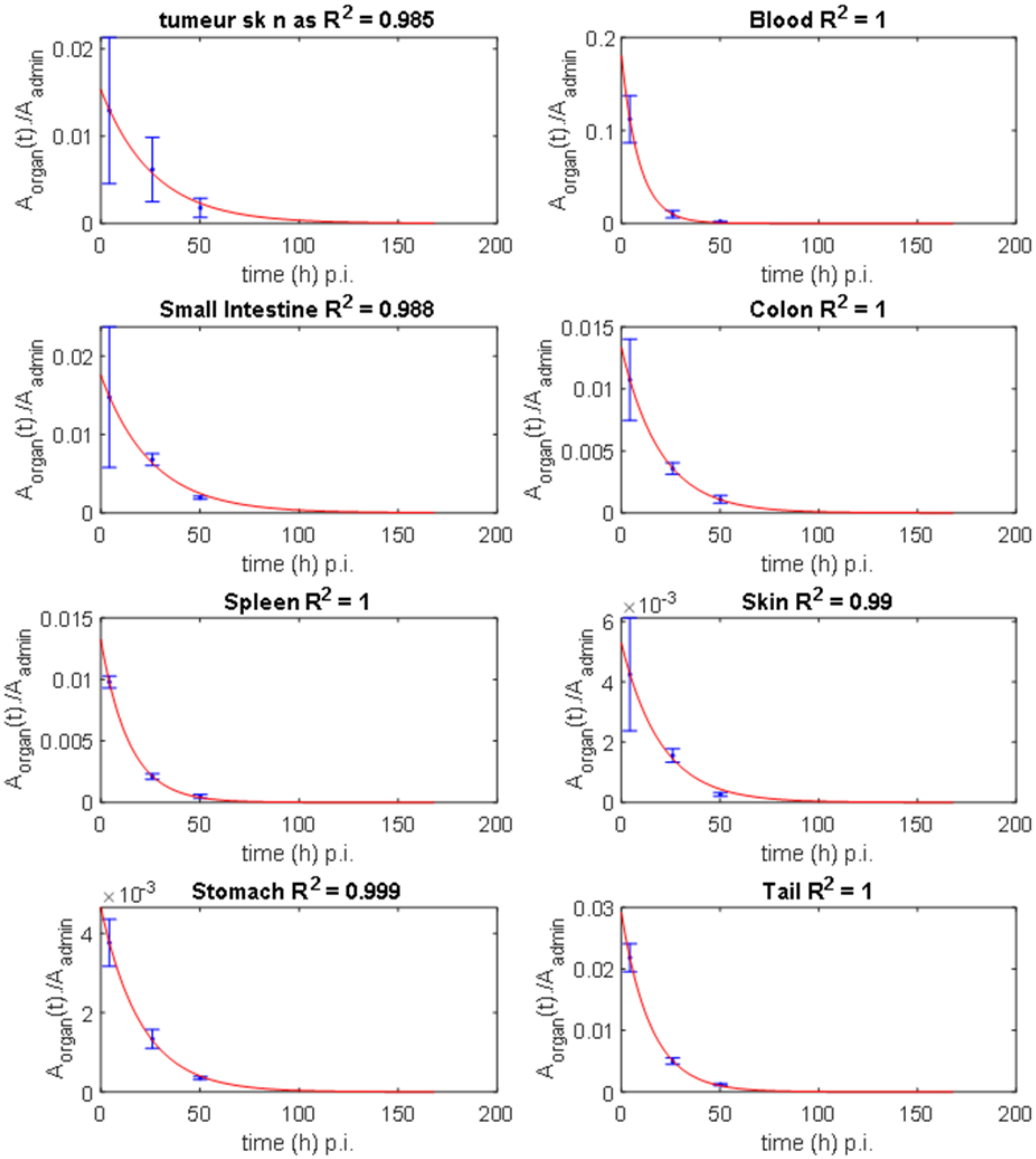
tumeur sk n as R2 = 0.985
Blood R2 = 1
Small Intestine R2 = 0.988
Colon R2 = 1
Spleen R2 = 1
Skin R2 = 0.99
Stomach R2 = 0.999
Tail R2 = 1
Aorgan(t) /Aadmin
time (h) p.i.
×10-3

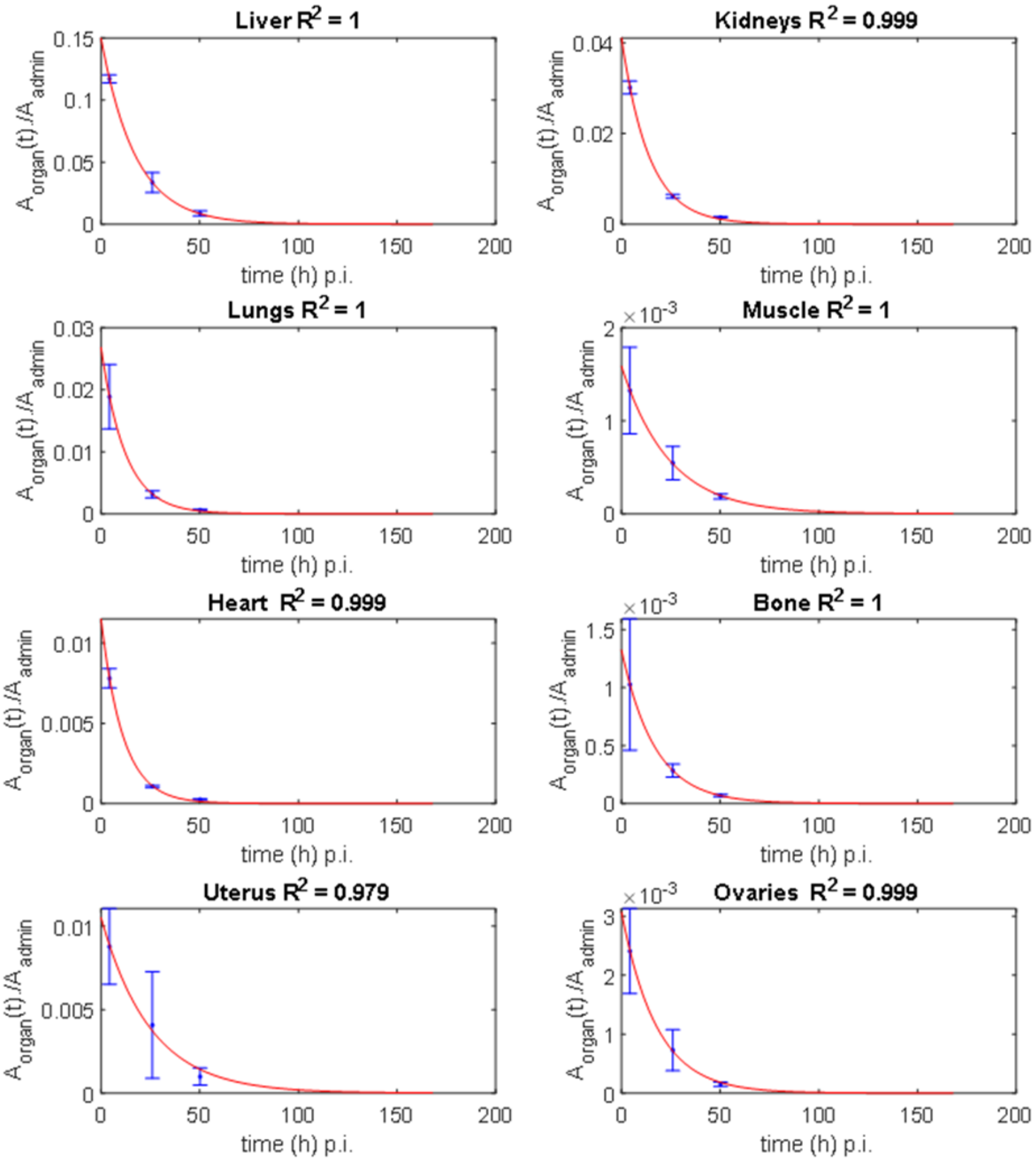

Figure S3. Normalized time-activity curves for the considered source organs (experimental data in blue with respective ± SD interval). Red lines represent mono-exponential fitting curves obtained for source organ nTACs. The coefficient of determination (R^2) of the fit in respect to experimental data (blue dots) is also reported.
